# Traffic Congestion Analysis Based on a Web-GIS and Data Mining of Traffic Events from Twitter †

**DOI:** 10.3390/s21092964

**Published:** 2021-04-23

**Authors:** Juan Salazar-Carrillo, Miguel Torres-Ruiz, Clodoveu A. Davis, Rolando Quintero, Marco Moreno-Ibarra, Giovanni Guzmán

**Affiliations:** 1 Comisión Nacional para el Conocimiento y Uso de la Biodiversidad, Mexico City 14010, Mexico; jsalazar@conabio.gob.mx; 2 Instituto Politécnico Nacional, CIC, UPALM-Zacatenco, Mexico City 07320, Mexico; rquintero@ipn.mx (R.Q.); mmorenoi@ipn.mx (M.M.-I.); jguzmanl@ipn.mx (G.G.); 3 Computer Science Department, Universidade Federal de Minas Gerais, Pampulha, Belo Horizonte MG 31270-901, Brazil; clodoveu@dcc.ufmg.br

**Keywords:** volunteered geographic information, crowdsourcing, spatiotemporal analysis, support vector regression, geographic information system, twitter

## Abstract

Smart cities are characterized by the use of massive information and digital communication technologies as well as sensor networks where the Internet and smart data are the core. This paper proposes a methodology to geocode traffic-related events that are collected from Twitter and how to use geocoded information to gather a training dataset, apply a Support Vector Machine method, and build a prediction model. This model produces spatiotemporal information regarding traffic congestions with a spatiotemporal analysis. Furthermore, a spatial distribution represented by heat maps is proposed to describe the traffic behavior of specific and sensed areas of Mexico City in a Web-GIS application. This work demonstrates that social media are a good alternative that can be leveraged to gather collaboratively Volunteered Geographic Information for sensing the dynamic of a city in which citizens act as sensors.

## 1. Introduction

Nowadays, mobile computing plays an important role to characterize the behavior, relationships, and dynamics of human being activities in big cities. Particularly, the development of applications oriented towards sensing specific tasks with the use of smartphones is increasing day-by-day to improve the well-being of the citizens [1]. Different techniques to sensing human activity in the geospatial context have been proposed [2]. Those present a shared and collaborative approach, using communication networks to collect data about diverse phenomena in a relatively short time and saving resources in specialized infrastructure. 

Nowadays, user-generated content is one of the most common and powerful sources to compile spatially referenced data as taking people as sensors worldwide. Thus, there are different platforms, websites, social networks that are used to comment or post activities, situations, or feelings, share multimedia, and use location-based services to inform and influence everyday life. New tools to gain insight into different forms of data that are themselves the result of the digital revolution, the growth of the Internet, and, significantly, mobile devices [3].

The approach coined as Volunteered Geographic Information (VGI) is intended to generate geospatial data in which human beings are conceived as “sensors”, and they are the main generation source. Thus, various public and open online applications, publications on social networks, as well as specific sensors embedded into mobile devices are used to create and share these compiled geospatial data by the citizens. VGI has changed paradigms in which information is created, shared, used, and experienced, with important implications for geospatial data applications, including emergency management, traffic congestion, air pollution, energy consumption, urban planning, crime rate, climate change, and energy consumption, among others. Thus, citizen-sensors were defined by [4] in order to compare humans to “intelligent, mobile sensors”, related directly to VGI for highlighting the concept of user-generated content attached with geospatial information (e.g., location, place names) in the form of geo-tags or coordinates. No matter what people, services, devices, and sensors are sensing (e.g., noise, air quality), the spatiotemporal context helps in the understanding and interpretation of the collected data [5]. The term geocrowdsourcing [6], or simply crowdsourcing [7], is widely used in Geographic Information Systems (GIS) for urban computing, which involves the collection of geospatial information performed by an undefined network of people.

Many platforms and systems have been designed to collect big datasets to represent the behavior and particular characteristics from different phenomena of diverse contexts [8]. Pervasive systems are oriented towards improving sensing approaches, providing more descriptive geospatial information that can be collected by streaming in real-time. In this position, dwellers of urban spaces play a fundamental position to sense the pulse of the cities. Thus, there is a widespread state-of-the-art to comprehend different relationships and behaviors associated with human beings to describe the urban dynamics. User-generated traffic information in mobile communication networks is frequently used as a data source. This information content can be used to generate better models of activity and mobility [9].

According to [10], traffic congestion is a problem that directly affects big cities. Different approaches to describe this state have been deeply analyzed. For instance, information obtained from mobile devices to characterize the traffic congestion based on clustering the road flow, according to different values and considering the human judgment is described in [11]; other approaches are focused on digital image processing [12], physical sensors installed in the streets [13], and pattern recognition techniques [14].

On the other hand, social media is a type of VGI platform, with fast information sharing facilities and a fast speed of online communication, as well as a large volume of people engaged with social networks such as Facebook and Twitter, enabling further communication and providing alternative means for information dissemination [15]. Studies have demonstrated that there is a strong connection between social relationships and geography, considering the data from Facebook [16]. Thus, people that interact daily almost always live near each other, and each user has at least 10 friends with shared locations.

In this paper, a methodology to geocode traffic-related events from the Twitter streaming, as well as how to use the geocoded information in order to gather a training dataset for applying a Support Vector Machine method and build a prediction model, is proposed. This model provides spatiotemporal information on traffic congestions with a spatiotemporal analysis. Moreover, the spatial distribution of the traffic-related events is represented by heat maps, which describe the concentration or density of the traffic congestion in a Web-GIS application. The results have been evaluated by applying precision and recall measures.

The paper is organized as follows: Section 2 presents the state-of-the-art in this field. Section 3 describes the methods and materials that comprise the proposed methodology to analyze traffic congestion and the behavior characterization by traffic-related events. Section 4 depicts the experimental results, applying the proposed methodology, and the evaluation of the proposed prediction model. Thus, Section 5 presents a discussion concerning the analysis of the results and performance of the methodology. Finally, Section 6 outlines the conclusion and future work.

## 2. Related Work

In the state-of-the-art, there are several approaches oriented towards obtaining georeferenced information from social media to process and visualize geographic in-formation on the Web, as well as applying Machine Learning algorithms to build prediction models that describe the behavior of different phenomena that impact cities.

For example, in [17] a model to predict traffic congestion is proposed. This approach consists of applying a deep learning technique, which compounds extracted data from Twitter with climate and transit data. It supports a Long Short-Term Memory, which is a deep bidirectional method to predict data. Thus, the model is trained by applying a stacked autoencoder architecture, generating test and training data sets with data regarding Twitter, traffic congestion, and climate. Moreover, a case study of Saudi Arabia to detect event-related to traffic congestion was proposed. The approach is based on the Apache Spark system and clustering methods to generate eight classes that represent different states or events [18].

On the other hand, a case study of Washington DC to model the traffic phenomenon was presented in [19]. This approach consists of establishing keywords, considering “traffic safety” data, which were obtained from Twitter for the last four years. Sentiment analysis was implemented over the tweets’ corpus, in which an extraction–transformation–cleaning (ETL) process was applied. Thus, some citizens’ faiths and moods were computed to determine the importance to reduce the deadliness, vehicular security issues, and the application of transit and official policies. Moreover, six high profiles were established such as drivers without seat belts, spoiled control, top speedup limits, distracted conduction, younger chauffeurs, and very mature drivers.

A technique to analyze different patterns associated to work and rest activities and how traffic congestion influences these states during different moments of the day is described in [20]. The authors proposed to determine these patterns by extracting information from Twitter. Later, sentiment analysis was also applied taking into consideration tweets that reflected the moods of the citizens. The study demonstrated that the activity of the dwellers during the day determines the traffic flow to the next day. In other words, depending on their rest or activity in the previous night, the traffic flow is represented in the roads. Thus, [21] proposed an application to sense traffic congestion by mining continuously the Twitter streaming. The collected tweets are clustered by applying a feature-extraction algorithm, considering the occurrence and frequency of the traffic congestion in specific areas. This research work realized a comparative analysis to assess different clustering techniques regarding the application performance.

On the other hand, an analysis performed in Valencia, Spain to determine the spatial traffic dispersion is presented in [22]. The analysis consisted of classifying diverse avenues over the city, defining the congestion flows with spatiotemporal data, and taking into consideration trips in rush hours, and the number of lanes per street. The outcomes demonstrated that both variables impact only in certain segments of streets, and only an adjustment in the real conditions determines the vehicular flows to specific areas within the city.

According to [23], traffic states are usually not perfectly measured and are everywhere. Thus, an estimation is required from local and noisy sensor data. One of the most widely applied estimation methods is the Lighthill–Whitham and Richards (LWR) model with an extended Kalman filter (EKF). An important disadvantage of the EKF is that the method is too slow to perform in real-time on large networks. To overcome this issue, the authors proposed a novel localized EKF (L-EKF) algorithm. The logic of the traffic network is used to correct only the state in the vicinity of a detector.

According to the state-of-the-art, public and open applications, as well as centrally devoted platforms focused on collecting, storing, and sharing VGI, have been proposed [24]. In this context, collaborative applications oriented towards improving the social civic participation in activities associated with compiling data to enhance diverse electronic services and generate diverse data repositories for the well-being of big cities were proposed in [25]. Moreover, [26] presents a good comparative and analysis concerning VGI products that have been created with the design of systems focused on processing and assessing user-generated data.

Many works related to obtaining VGI from social networks have been proposed. In other cases, approaches to recognize geospatial facts that occur in certain locations are presented in [27]. A methodology for geocoding tweets to develop a map based on these given geographic references is described in [28]. Moreover, an approach in which citizens were adopted as “human-sensors” to detect earthquakes based on publications of Twitter that reflected this phenomenon is proposed in [29]. According to [30], human mobility plays an important role in the study of traffic forecasting and the engineering of smart cities. Approaches to face how to model traffic flows in big cities, as well as the development of transportation infrastructure for the citizens and visitors, is a very timely challenge. The flow or congestion is classified into two types: continuous or discontinuous. Thus, the usual traces of the citizens describe a natural behavior with repetitive patterns, which describe a continuous flow. The discontinuous flow is defined by random events such as vehicular accidents, climate situations, among others. In this context, insights to handle discontinuous flows are very important as well as the detection of these kinds of events allows us to determine efficient manners to control traffic congestion.

In [31], an approach to mine tweet texts for extracting incident information on highways as an efficient and cost-effective alternative to existing data sources is proposed. It consists of a crawling process and filtering tweets that are freely accessible by citizens. So, keywords’ thesaurus and the combinations of these terms allow determining traffic events from the data acquisition process. Therefore, a post is handled as an n-dimensional vector according to the characteristic slot that is built by the thesaurus and clustered into traffic-related events or not. In this stage, the corpus of tweets was previously georeferenced to find the geospatial position. In the same context, the problem of interpreting tweets that describe traffic-related events, which are distributed by government agencies in charge of road networks or by news agencies is presented in [32]. The contribution is an automatic tweet interpretation tool, based on Machine Learning techniques, achieving good performance for traffic-related tweets that are distributed by traffic authorities and news agencies. Ref. [33] proposed a methodology based on user relationships to infer the location of messages on Twitter. A network is created considering the follower–following relationships, starting from the known locations of users in the network and inferring the location of others.

Research proposals to determine the performance of traffic models have been deeply studied. For instance, comparisons and tests regarding optimization techniques such as the analysis of genetic algorithms are presented in [34]. The authors proposed the deterministic Nelder-Mead method, with the application of a stochastic algorithm, and the computation of the entropy to determine values of real traffic flows in different roads. The assessment of the model was carried out considering different transit data sets to evaluate the precision of the results, and how the time to resolve is optimized. Moreover, a traffic simulator application to reproduce traffic information behavior was proposed in [35] to evaluate usual areas with a high density of vehicular traffic.

The advances in mobile computing and social networking services enable people to probe the dynamics of a city. In [36], a method based on crowdsensing mixed with information on human mobility and social media to detect and describe traffic anomalies is presented. The detection of events was modeled by a sub-graph, which represents a road network, where drivers’ routing behaviors significantly differ from their original patterns.

Other approaches are focused on identifying geographic features from the text, without using Natural Language Processing (NLP). In [37], a method to identify traffic events and conditions in Twitter, geocode them by using a GEODICT gazetteer, and display them on the Web in real-time was proposed. The GEODICT gazetteer is a data source where toponyms (place names) are associated with concepts and their geographic footprint. Thus, it is a free repository that contains basic yet precise information such as multilingual labels, and administrative boundaries polygons, among others, that can be customized. Preliminary results showed that the method is able to detect neighborhoods and thoroughfares with a precision that varies from 50 to 90%, depending on the number of places mentioned in the tweets. 

Traffic prediction models, transportation planning, and services centered on smart cities are deeply aligned with human mobility. In [38] a design to implement an application of automatically catch citizen perceptions is proposed. The main conception is focused on generating a mobile space to compile user-generated content. Assumptions concerning the feasible information and the qualitative data value were analyzed. The approach was intended for urban practitioners and how to exploit these human mobility data. Twitter was used to mine messages, taking into consideration those that describe traffic situations. Subsequently, a natural language method to classify the tweets automatically was implemented.

Machine Learning and probabilistic techniques have been also used in network traffic analysis. In [39], a statistical approach based on R software and GPS traces of vehicles was proposed. The traces were mined to extract the outlier traffic pattern. The urban was divided into a grid, and the road infrastructure was organized as segments of a graph. The congestion level was determined by analyzing the visits for each vehicle using the GPS trace data. Moreover, [40] introduced a traffic model based on the probabilistic topic method to describe the traffic states for a variety of roads. Thus, a technique for detecting traffic incidents from probe-cars by identifying unusual events that distinguish incidents from spontaneous congestion was presented. The approach detected successfully between anomalous car trajectories and the more usual, slowly moving traffic patterns. In [41], a fast, non-convex multi-task sparse feature-based learning method to define a set of shared features in traffic data was proposed. The method can learn features belonging to each task as well as the common features shared among tasks. In [42], various models and approaches using soft computing techniques to tackle the problem have been developed. Major soft computing approaches for this purpose are Fuzzy Approaches, Neural Network and Genetic Algorithms, Petri Nets, and so on. Moreover, multi-agent systems were highly applicable in this approach. Ref. [43] proposed a system for individual trip planning that incorporates future traffic hazards in routing. The traffic conditions were computed by spatiotemporal random field on a stream of sensor readings. The traffic flow in areas with low sensor coverage was estimated by Gaussian Process Regression. Moreover, [44] presented an application based on a supervised statistical learning technique, which consists of a prediction model implemented in a Support Vector Regression (SVR) to predict freeway traffic flow under both typical and atypical conditions.

## 3. Method and Materials

The proposed approach provides a general framework, which is composed of the following stages: (1) data acquisition and information analysis, (2) the creation of dictionaries and equivalents, (3) gazetteer division, (4) standardization, (5) the identification and location of traffic-related events, (6) the generation of a prediction model, and (7) the visualization of traffic-related event prediction.

**Figure 1:**
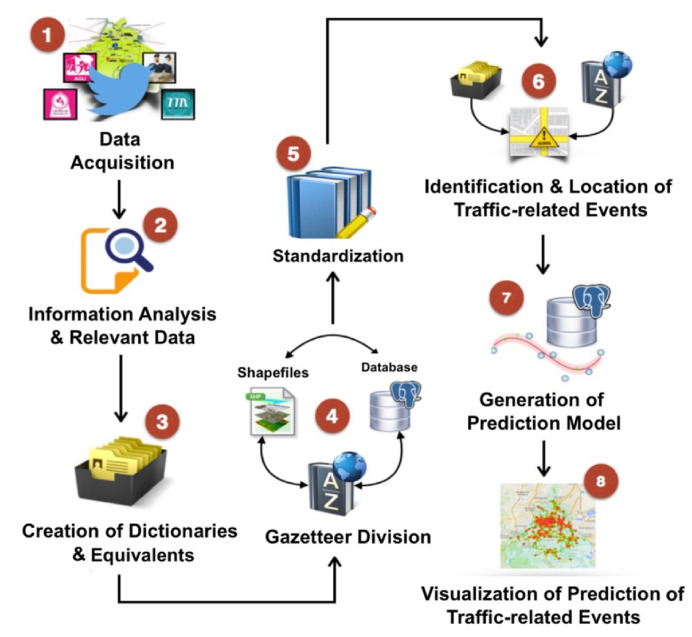
The general framework of the proposed approach.

The geocoding process is embedded from stage 1 to stage 6. The following stages are in charge of generating the prediction model and the visualization of traffic-related events that were predicted by the model. Figure 1 depicts the main phases that compose the general framework. This research work is oriented towards initiating from the data acquisition to the visualization of the prediction of traffic-related events. Thus, this Figure offers a general conceptualization of the work proposal.

Thus, the data acquisition and its geocoding process with the prediction model related to traffic events, as well as the visualization of results, are described in detail in the following Sections.

### 3.1. Geocoding Traffic-Related Events from Tweets

In this stage, both automatic and manual steps are required with the purpose to search the following geographic elements on Twitter: streets, public transportation stations, local areas, among others. Thus, the GeoNames gazetteer and a tweet dataset were used to initiate this process.

GeoNames is a dataset that contains 36,236 streets from Mexico City and a minimum of 150,000 street segments. It describes the features of geographic objects that contain information in the Spanish language. For this reason, any field could contain an empty value, or, the label “NO NAME”, starts with one of the next abbreviations: ‘rd, ‘st’, ‘ave’ (road, street, and avenue); and finally, the charset includes low and upper case and special characters (á, é, í, ó, ú). 

#### 3.1.1. Data Acquisition and Information Analysis

An analysis to select particular accounts from the tweet stream was made. Many tweets talk about traffic-related events, but it is important to determine and identify tweets that could describe an event using the information of crowded streets, common nicknames, short names, famous places, and historical monuments. With this dataset, an algorithm for computing the most common words was implemented, the output of this algorithm is a list of the most common N-grams. Thus, the operation of the algorithm is described as follows:

(a) Read a tweet (*t*) from the tweet dataset (*TD*).
(b) Define the value of *N* for the N-gram computing process.
(c) Compute the occurrence of each N-gram.
(d) Apply a sort descending algorithm.
(e) Select the most repetitive N-grams according to a threshold.

**Figure 2:**
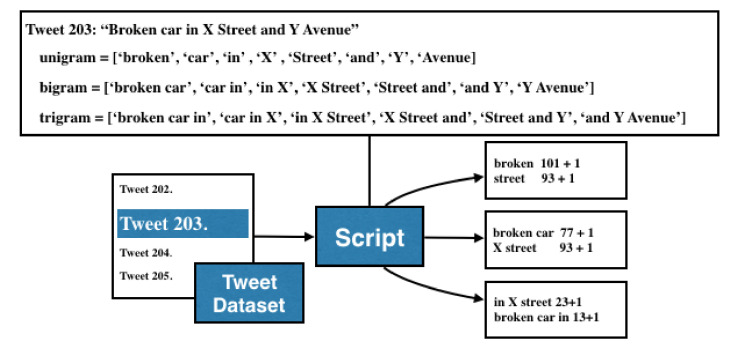
Computing of the lists with most frequent N-grams by the script.

A practical value for this threshold is 100. So, to reduce the computational complexity of this process, a restriction was established so that only continuous slices of N-words can be valid. In Figure 2 we illustrate the operation of this algorithm:

The results obtained from the N-grams are the following: 150 common traffic-related events, 69 common nicknames; 34 common abbreviations; 456 common streets,135 common hashtags; 65 common buildings, places and, monuments; and 26 similar combinations of prepositions that were identified by hand. 

#### 3.1.2. Creation of the Dictionaries and Equivalents

**Figure 3:**
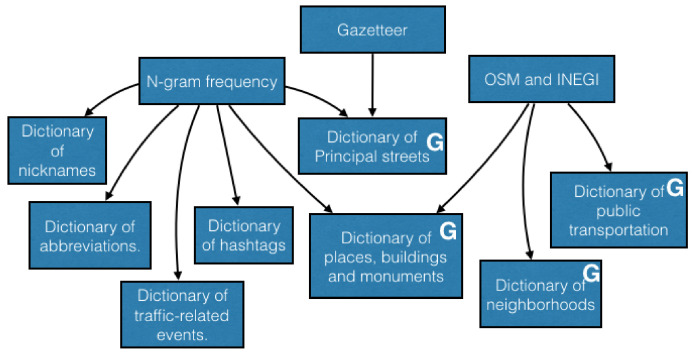
Creation of dictionaries based on information analysis.

By considering information hold of the preceding stage, figures the National Institute of Statistics and Geography (INEGI) of Mexico were used to bring about and enrich some glossaries and the bulletin (see Figure 3). The proposed glossaries are the next ones: short forms, alias, octothorps, movements, urban transportation service, main roads (those that show up in tweets and are present in the bulletin), locations, constructions, and markers, and areas. Some dictionaries have a geographical feature, that is required to dimensionally sketch earthly elements. Thus, these glossaries are identified by the letter “G” in the blocks of Figure 3, and they were called dictionaries of geographic elements. On the other hand, a glossary of geographical relationships might have been set up; however, in this method, the traffic-related events are classified by using the number of geographic elements that were identified in the tweets.

Regarding the equivalents, it is habitual that avenues shall be signed for over more than one known name. In brief, Mexico City has 31 artery cuts and two peripheries covering over more than 10 thousand kilometers all around the city. Cuts and peripheries modify their names as they reach different streets and crossed roads. Thus, they are occasionally known with their most known name, or with the name of a specific segment (popular name), or just by putting names together (principal name + second name). Yet, all these different names are accepted; for this reason, all the different options have to be explored in the tweets. With the idea of looking for heterogeneity regarding the names, a glossary of equivalent artery cut names was considered to clarify the named collection.

#### 3.1.3. Gazetteer Division

According to information analysis, commonly a few streets have the most influx-related events. With the N-gram densities, just 19 percent of the roads that show up in tweets of the whole bulletin was actually localized. Thus, the bulletin is divided into two parts:

- One of them contains the known streets frequently mentioned in tweets that exist in the bulletin (some ways shafted in tweets are in the suburbs of Mexico City, so they were left out).
- The second one contains the rest of the streets. Although the cut did not make the precision better and recall of geocoding, the achievement of the identification as well as the location steps had an increase by using this division.

#### 3.1.4. Standardization

The standardization process was improved by considering dictionaries of non-geographic elements, such as abbreviations, nicknames, and hashtags. In the proposed gazetteer, the names of streets contained in the geographic dictionaries present abbreviations, capitalized names, with stressed marks on the names, gaps between rows, etc. For this reason, changes each street name to lowercase, and the accent marks are removed (TALISMÁN to talisman). Additionally, the glossary of common short forms is used to substitute the complete sentence (talisman St.—talisman street). At last, blank gaps and ways with default values are eliminated.

Other issues that arose in tweets are related to chains and referents to other versions, alias, misplaces, and, octothorps (e.g., http://t.co/hAN0K0WS (accessed on 27 July 2020), @OVIALCDMX, “The angel”, “circuito interior street”, #insurgentesavenue). Thus, with the idea to solve these new features, the glossary of alias, and octothorps were intended to substitute those with the name given by the government in tweets (for instance, “The angel”—“angel of independence” and #insurgentesavenue—“insurgentes avenue”). Chains and referents to other versions were removed. The misplaces topic is never treated in this work.

Additionally, in posts as well as in gazetteers, the stop words have to be factored out. These words do not give additional meaning to the sentence. Moreover, these words are very common in a language (articles, pronouns, and prepositions). As we do not have a list of stop words for using in Natural Language Processing. This is why the stop word list has been sketched by the Natural Language Toolkit Library [45].

#### 3.1.5. Identification and Location of Traffic-Related Events

It has been carried out the identification of geographic elements only by using all the glossaries of geographic elements. Accidents, traffic-related tweets from chosen accounts often describe either bad or good traffic conditions. As an example, incidents like accidents are known as “car crashes”, “rollovers”, “emergency services”; bad conditions are “slow displacement”, “blocked roads”, “settlements”, etc.; and good conditions are denoted as “yet moving”, “fluid displacement”, etc.

Even when there is a good quantity of signs, inquiries, and safety suggestions, they are easily factored out due to the fact of lack of any geographical information as well as the short length of posted tweets.

When we consider an amount of a tweet corpus and the N-gram frequency analysis, a vehicle incident is contemplated as a collision or incident on the public road a specific punctual location involving at least one geospatial object. For instance, “a road traffic accident at x street and z street”, “a broken traffic light at crossroads the intersection of x street and y street”, “overturn vehicles in front of x subway station”, etc. The explanation of a false or true state is set by the verified state of affairs of a road portion. The aforementioned descriptions, often one, two, or three or n geospatial objects are taken into account. For instance, “a traffic jam on x street between y street and z street”, “a good circulation of cars on x street from y street to z locality”, “rush hour in x street on z locality”, “rainfall over x neighborhood”, among others [46].

**Figure 4:**
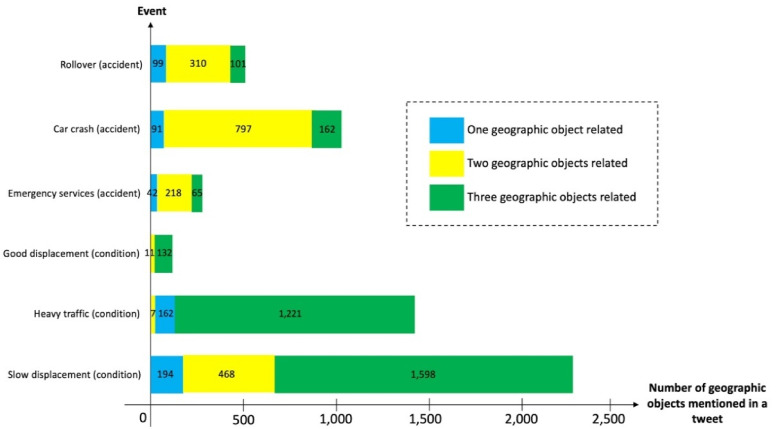
Examples of common traffic-related events that appear in tweets and the number of geographic elements detected with the proposed approach.

A fragment of the list of accidents and conditions that were identified with the proposed method is presented in Table 1. Since tweets can only contain 140 characters, it is difficult to post a mention, a link, a traffic-related event, and more than three geographic elements. Therefore, the number of geographic elements included in the tweet has a strong relationship with the kind of traffic-related event (see Figure 4).

Instead, each glossary of geospatial objects has a basic geographic depiction (punctual, arc, area). Therefore, the glossary of mass transit is rounded out by a set of punctual objects; the glossary of streets is distinguished by a set of arcs, and the glossaries of districts, sites, buildings, and memorials are illustrated by areas. Therefore, a collection of geographic primitives was obtained by searching for geographic elements from dictionaries in tweets.

Taking for granted that there are not 1, 2, or 3 testimonials to places in a tweet, the number of correlations (relationships) that can occur amid them is calculated by Equation (1).

(1) $$CR_{q}^{p}=\left(\frac{q+p-1}{p}=\frac{\left(q+p-1\right)!}{p!\left(q-1\right)!}\right)$$

where *q* is the number of possible objects to be chosen; in this respect, punctual, arc, or area, and *p* is the number of objects that were retrieved. Hence, for one object: [(punctual), (arc), (area)], with two-spotted components: [(punctual, punctual), (punctual, arc), (punctual, area), (arc, arc), (arc, area), (area, area)], and three spotted components: [(punctual, punctual, punctual), (punctual, punctual, arc), (punctual, punctual, area), (punctual, arc, arc), (punctual, arc, area), (punctual, area, area), (arc, arc, arc), (arc, arc, area), (arc, area, area), (area, area, area)]. A lot of these connections of the geospatial basics are not exact regarding the location or prevalence in tweets is not excessive; therefore, they can be thrown away [46].

In this approach, the relationships that were considered are the following: [(*pt*, *l*), (*l*, *l*), (*pt*, *pt*, *l*), (*pt*, *l*, *l*), (*l*, *l*, *l*), (*l*, *l*, *p*)], where *pt*, *l*, and *p* represents a point, line, and polygon respectively. These relationships were recognized in the corpus obtained from the dataset; the hypothesis of each relationship is conceived as follows:

- (*pt*) describes an event in an urban transportation or service station.
- (*pt*, *l*) is an arterial road segment restriction in front of an urban transportation service station.
- (*l*, *l*) describes an event in an arterial road crossing point.
- (*pt*, *pt*, *l*) describes an arterial road segment restriction outlined by two urban transportation service stations.
- (*pt*, *l*, *l*) describes an arterial road segment restriction outlined by other artery and urban transportation service station.
- (*l*, *l*, *l*) describes an arterial road segment restriction delimited by two arteries.
- (*l*, *l*, *p*) describes an arterial road segment delimited by an artery and place, building, or historical monument.

On the other hand, it is important to define a set of three spatial operations to compute the final result of applying these assumptions. The spatial operations are described as follows:

1. Determine the arterial road intersection;
2. Determine the polygon, or the line, that is closest to the point of interest;
3. Determine the bounding box of the line portion.

To apply these spatial operations, we implemented scripts using the PostGIS spatial functions: ST_Intersection, ST_ClosestPoint, ST_Envelope, and ST_ConvexHull. Thus, the final result represents the location of the traffic-related event. In Table 2, the procedure to determine an (*l*, *l*, *l*) relationship is described.

### 3.2. Generation of the Prediction Model

From the geocoding method that was described in Section 3, a collection of geocoded tweets was obtained. This collection is used to generate a training dataset for the Machine Learning method (Support Vector Machine). There are some features that were considered for this collection. Moreover, the selection of features in a Support Vector Machine determines the success of the learning approach. However, the best way to select the most relevant features is manually, based on the deep knowledge about the learning problem and the meaning of each feature.

A training dataset is composed of an input that is an *n*-dimensional vector and the output that is a real number. In this case, the *n*-dimensional vector contains the features that better represent the collection of tweets, and the output is the location where the geocoding method found the traffic-related event. Due to the tweet restriction regarding 140 characters, it is difficult to obtain a vector with multiple features to create a training dataset. Thus, only features of traffic-related events that happened in the analysis were taken into consideration. Table 3 presents the spatiotemporal features and the possible values that these events could take for the training dataset.

On the other hand, some features were discarded because they added noise to the results. In addition, geographic features over tweets are not usual; only a small quantity of tweets contains the neighborhood, zip code, or nearby places. Therefore, a supervised method cannot use information from the output for inferring the values of the selected features.

Thus, the traffic-related events were classified according to their district in which a prediction was correlated with each district. In this case, the precision and recall measures increase considerably because the division of the training dataset per district reduces the possibility of finding vectors with similar values that belong to distant locations. Thus, Mexico City is composed of 16 districts; consequently, there are 16 training datasets, which define 16 prediction models.

#### 3.2.1. The Support Vector Regression Method

The aim of the following mathematical presentation is only to clarify some of the concepts used in the work, how they are computed, and where they come from.

Thus, consider a training dataset of size *n*, denoted {(*x_1_*, *y_1_*),…, (*x_n_*, *y_n_*)}, where *x_i_* ∈ *R^k^* is the input space of the sample, and *y_i_* ∈ *R* is a related numerical value that represents the output of each sample. The purpose of the regression problem is to determine future values with precision by means of a function modeled. The general function of SVR takes the form of Equation (2).

*y*(*x*) = ω^T^ Φ(*x*) + *b*, (2)

where ω ∈ *R^k^*, *b* ∈ *R*, and Φ are the non-linear transformation from *R^k^* to high dimensional space. Φ transformation is a primary characteristic of Support Vector Machines (SVM), it is called *kernel trick*. The main idea of this transformation is to move to another higher dimensional space when the samples are non-linearly separable. Therefore, the goal is to find the values of ω and *b* determining the values of *x*, minimizing the regression risk (see Equation (3)).

(3) $$R_{reg}\left(y\right)=C\sum_{i=1}^{n}E_{\varepsilon }\left(y\left(x_{i}\right)-t_{i}\right)+\frac{1}{2}{\left|\omega \right|}^{2},$$

where *E*_ε_ is a cost function, and *C* is a penalization constant. The value of the cost function (Equation (4)) is zero if the absolute difference between the prediction *y*(*x*) and the target *t* is less than ε, where ε > 0. Otherwise, the cost is |*y*(*x*) − *t*| − ε.

(4) $$E_{\varepsilon }\left(y\left(x\right)-t\right)=\left\{\begin{array}{ll} 0, & if\;\left|y\left(x\right)-t\right|<\varepsilon_{i}\\ \left|y\left(x\right)-t\right|-\varepsilon & Otherwise \end{array}\right.$$

The optimization problem embedded in SVR needs slack variables in order to improve the accuracy of the model. In SVM, even if a linear separation is achieved in the feature space Φ(*x*), this separation of the training data can lead to a poor generalization; therefore, it is necessary to find a way to modify the SVM so as to allow some of the training points to be misclassified. Thus, slack variables modify the approach allowing points to be on the wrong side of the margin boundary, but with a penalty that increases with the distance from that boundary.

In SVR, for each data point *x_n_* two slack variables are added, ξ ≥ 0 and $\hat{\xi }$ ≥ 0. The first one where ξ > 0 corresponds to a point for which *t_n_* > *y*(*x_n_*) + ε. In the second, $\hat{\xi }$ > 0 corresponds to a point for which *t_n_* < *y*(*x_n_*) − ε.

The introduction of slack variables in the case of SVR allows points to lie outside the ε-insensitive region that is commonly called ε-tube, and the corresponding conditions are defined in Equations (5) and (6).

*t_n_* ≤ *y*(*x_n_*)+ ε + ξ*_n_* (5)

(6) $$t_{n}\geq y(x_{n})-\varepsilon -{\hat{\xi }}_{n}$$

The error function for SVR can now be defined as in Equation (7).

(7) $$R_{reg}\left(y\right)=C\overset{N}{\underset{n=1}{\sum }}\left(\xi_{n}+{\hat{\xi }}_{n}\right)+\frac{1}{2}{\left|\left|\omega \right|\right|}^{2}$$

This error must be minimized subject to the constraints ξ > 0, $\hat{\xi }$ > 0 defined in Equations (5) and (6). Thus, the Lagrange multipliers $a_{n}$(ε + ξ*_n_* + *y_n_* − *t_n_*) ≥ 0, and ${\hat{a}}_{n}$ (ε + $\hat{\xi }$*_n_* − *y_n_* + *t_n_*) ≥ 0 are used to compute the optimization.

Now, the value of ω in terms of the Lagrange multipliers is described by Equation (8).

(8) $$\omega =\overset{n}{\underset{i=0}{\sum }}\left(a_{n}-{â}_{n}\right)\Phi \left(x_{n}\right)$$

The *b* variable is computed by applying Karush–Kuhn-Tucker (KKT) conditions, and Φ is the kernel trick value. It implies that the product of the Lagrange multipliers and constraints must be equal to zero (see Equations (9)–(12)).

*a_n_*(ε+ ξ*_n_* + *y_n_* − *t_n_*) = 0 (9)

(10) $${\hat{a}}_{n}(\varepsilon +{\hat{\xi }}_{n}-y_{n}+t_{n})=0$$

(*C* − *a_n_*) ξ_*n*_ = 0 (11)

(12) $$(C-{\hat{a}}_{n})\cdot {\hat{\xi }}_{n}=0$$

In this case, *b* is computed by applying Equation (13).

(13) $$b=t_{n}-\varepsilon -\omega^{T}\Phi \left(x_{n}\right)=t_{n}-\varepsilon -\overset{N}{\underset{m=1}{\sum }}\left(a_{m}-{â}_{m}k\left(x_{n},x_{m}\right)\right)$$

#### 3.2.2. The SVR Library

The Scikit-learn framework was used to compute these Equations. It is an open-source Machine Learning library that supports supervised and unsupervised learning. It also provides various tools for model fitting, predicting, cross-validation, data preprocessing, model selection and evaluation, and many other utilities.

The selection of this framework obeys the feasible mechanisms to introduce different programming languages. In this study, Python language was used to implement different algorithms, using different components embedded into the library such as regression and clustering algorithms, diverse classification techniques, and statistical methods. Moreover, the programs implemented into the Scikit-learn are suitable with the NumPy and ScyPy, which are scientific libraries of Python.

The most important methods and algorithms of the Scikit-learn as well as some libraries such as NumPy were programmed in Python language. The kernel of the techniques and Machine Learning algorithms were also programmed in Cython, in order to enhance the performance for the users. Moreover, the *libsvm* package contains the main regression algorithms such as Support Vector Machines. In the same case, the *liblinear* package stores the Logistic Regression and Linear Techniques. Other packages programmed in Python have also been integrated into the Scikit-Learn, for example, NumPy and SciPy oriented towards handling array vectorization, Pandas to control dataframes’ structures, and *matplotlib* and *plotly* to plotting and edition [47].

Regarding the conception of Python language, its conceptualization is focused on reusing code, with specific characteristics such as interoperability, readability, easy syntax, and definitions of concepts with simple code lines. It is considered as a high-level language, with a diverse mechanism to simplify the programming building in different development scales. Besides, Python is the most important language to develop fundamental applications oriented to data science.

Thus, SVR is used to obtain a reliable prediction model, and it requires two processes: training and testing. The first one uses a mandatory training for the SVR method in order to make future predictions using the training dataset. The second uses a test dataset to validate the prediction model; this dataset was taken from the training dataset.

The SVR library requires several parameters to establish the prediction model. The value of these parameters varies depending on the learning problem. The list of parameters is defined in Table 4. 

The parameters that were used to build the prediction model of traffic-related events with the highest precision are *C* = 1000, *gamma* = 0.04, and *epsilon* = 0.0004. These values were chosen manually by making *k*-fold cross-validation tests in order to ensure that the training dataset and the test dataset are isolated from the results.

The implementation of the prediction model and the simulations of the obtained predicted data were developed in Python language under the version 3.8.8, using Anaconda, which is a package management service. Additionally, it can be used to facilitate a development cycle and organize the code that is in development, in testing, and in production, without affecting non-development users. With labels, one can upload a file to a specific label so only users who put that label in the URL that they search are able to find it.

The training dataset was divided into input and output in order to use the SVR library and generate the prediction model. When the model is trained, the following process in the prediction model was tested using the test dataset.

- svr_rbf.fit(input_train, output_train);
- svr_rbf.predict(input_test).

## 4. Experimental Results

This section presents different results related to the proposed methodology in order to assess each stage by applying the methods to a particular case study.

### 4.1. The Empirical Data

After the cleaning process, the corpus has 65,250 posts on Twitter. This collection was performed from 7 July 2020 until 22 December 2020, and each tweet is “original”. It means that the corpus does not contain retweets, favorite marks, and tweets with blank spaces. The sources to collect each tweet of the corpus come from trustworthy Twitter accounts, which are related to official institutions and certificate communication services (see Table 5). The criteria that were used to select those accounts accomplish the following issues: account reputation, number of followers, tweets per day, date of creation, geographic location of the account, website of the account, government account.

Thus, the @Supervia_CDMX and @072AvialCDMX are Twitter accounts that correspond to government institutions. They are the accounts that more tweets provide to the corpus. There are important characteristics that allow us to validate the reputation of the account such as the description of traffic events is well-structured, good comprehension to post incidents, the number of followers is increasing, and the majority of its tweets can be georeferenced with good accuracy. Regarding @Trafico889 and @RedVialRC accounts, they are associated with radio broadcasting; so, data of climate conditions and traffic congestion are posted in their timelines, with the advantage that these tweets are also presented on their websites. Finally, @Alertux and @PolloVial accounts represent the user-generated content approach because they collect collaboratively volunteered data and make retweets from other Twitter profiles.

These empirical data have been preprocessing with natural language techniques, and the tweet dataset is volunteered information. In background mode, the collection is continuously sensed and stored in a spatial database within PostGIS in order to obtain more general information that in the future can help other projects to determine rates of air pollution, crime rates in particular areas, energy consumption, and environmental pollution, among others.

In addition, it is important to mention that the official government of Mexico City does not have any traffic information collection directly related to social networks in order to characterize and analyze the behavior of the traffic situation by means of a spatiotemporal approach. Thus, the empirical data collection was generated in order to represent traffic information provided by Twitter in which this phenomenon is not depicted in a map. Furthermore, the updating of this information is faster than classic cartographic techniques, and the integration with other Volunteered Geographic Information such as OpenStreetMaps and GeoWiki is feasible.

The novel contribution focuses on developing an approach with low cost in which the main collected information is provided by Twitter. Thus, the proposed empirical dataset makes a big difference from the conventional traffic data collection methods, which are based on roadside inductive loop detectors that are costly to deploy and maintain. Thus, social media is an alternative that can be leveraged to gather collaboratively Volunteered Geographic Information about the conditions of roads, streets, and avenues.

### 4.2. Evaluation of the Geocoding Method

In order to evaluate the accuracy of the geocoding method, a test dataset was put together using 652 geocoded tweets by hand. Thus, streets, public transportation stations, neighborhoods, places, buildings, and monuments were identified.

Moreover, the test dataset was compared with elements identified by the proposed methodology, and the *precision* and *recall* measures were also computed. The first evaluation consisted of comparing the baseline with only the gazetteer with part of the standardization, using only lowercase (see Section 3.1.4). The second one was compared with the baseline plus the full standardization. Finally, the third one used the baseline plus the standardization process plus the equivalent axis names.

Thus, the *true-positive*, *true-negative*, and *false-negative* values were used as parameters to compute the accuracy of the geocoding method, according to the proposed measures. In this case, *true-positive* values represent geospatial objects that were detected by the algorithm and these objects were also established as the gold standard metric in the design of the evaluation task. Concerning the *true-negative* values, they describe the geospatial objects that were detected by the algorithm; however, these objects are outside of the metric. Finally, the *false-negative* values represent the geospatial objects that correspond to the gold standard metric but they could not detect by the method. These calculations were realized to each tweet into the corpus and the mean was also determined. The outcomes regarding the evaluation measures are described in Table 6.

According to Table 6, it is appreciated that the proposed methodology has a precision and recall of 85% and 83, respectively, which is higher than the 39% and 31% obtained from the baseline. Thus, the best results were obtained with the definition of a dictionary of equivalent axes to identify geographic objects. Therefore, it implies a novel contribution to refine the performance regarding precision and recall.

**Figure 5:**
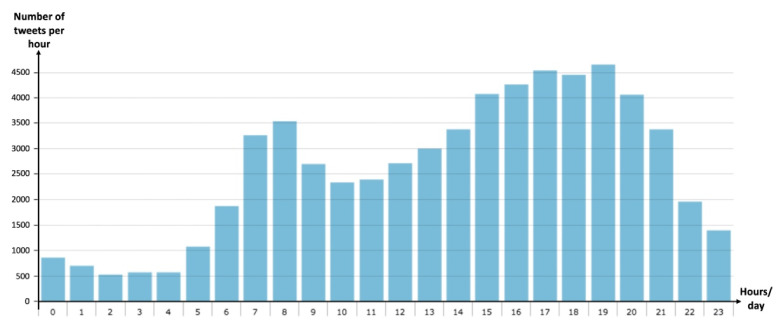
Relation of traffic-related events with Twitter.

From a temporal perspective, these results describe the relationship of traffic-related events in the real-world with Twitter. The number of tweets posted at 18, 19, and 20 h is higher. This is the time of the day with the highest level of participation that was found. Another relevant period is in the morning around 8 AM, with another important participation. Thus, this behavior corresponds to the rush hours in Mexico City (see Figure 5).

### 4.3. Visualization of Geocoded Tweets and the Prediction Data

**Figure 6:**
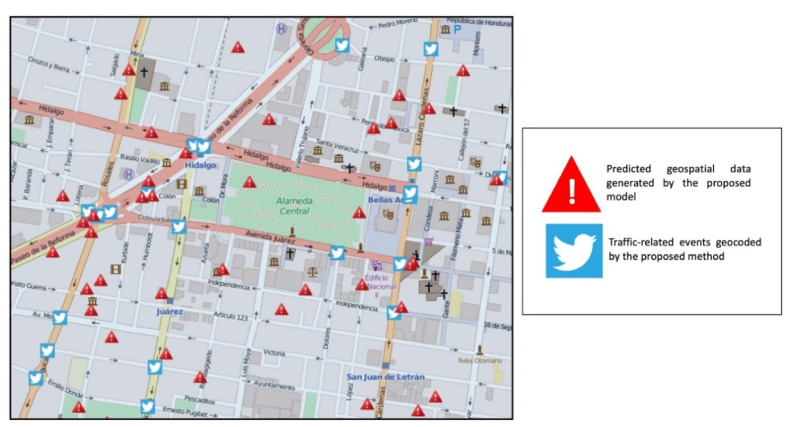
Comparison between predicted and geocoded traffic-related events.

In particular, the SVR method was implemented to define the prediction model, which requires two well-defined processes: training and testing. The first one uses a mandatory training dataset for the SVR method in order to make future predictions. The second uses the test dataset to validate the prediction model; this dataset was taken from the training dataset. Thus, the training dataset was divided into input and output in order to use the SVR library and generate the prediction model. Figure 6 shows a spatial comparison between the traffic-related events that were geocoded by the proposed method defined in Section 3.1 that are represented by the Twitter image with squares in blue color, and the predicted data that were directly generated by the prediction model based on the SVR, represented by triangles in red color. 

Concerning the spatiotemporal analysis of traffic-related events, it was generated by a collection of *n*-dimensional vectors with the same features of the training dataset input. This information was gathered by using a new prediction model with a training dataset that contains the *district*, *month*, *day*, *day of the week*, and *hour*. The characteristics are defined by vectors with the same structure that compose the input of the training dataset. The number of vectors that are sent to the training dataset varies for the regression analysis applying the SVR method in which the input of the training dataset is composed of the characteristics of the hour and day of the week, adding the spatial district characteristic that are represented as a categorized form.

The result of the output is the prediction model, which represents the number of accidents that could happen. With this number and the number of given constraints such as *hour*, *day of the week*, and *month*, a collection of *n*-dimensional vectors is obtained to send the traffic-related event to the prediction model.

**Figure 7:**
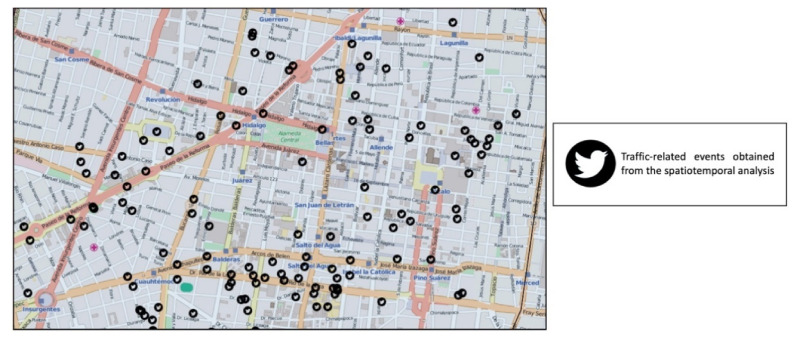
Traffic-related events that were obtained from the spatiotemporal analysis.

The values of the hour, day, and day of the week on each vector vary from the threshold from −1 to 1 to represent traffic values near the given constraint. Figure 7 depicts points in black color that represent the spatiotemporal analysis in which such points are traffic-related events. Thus, each vector that was sent to the prediction model is composed of the characteristics of time and space previously selected. Moreover, Figure 7 represents an example of the spatiotemporal analysis, taking into consideration the two components such as time and spatial location.

On the other hand, it is important to clarify that it is not possible to obtain the streets associated with traffic-related events because the general framework was intended to locate, analyze, and predict geospatial information that is represented by geographic points. After all, the geocoding process was designed to operate and validate only this type of geospatial representation. The methodology is not focused on retrieving linear geospatial objects, although this representation is used and defined in the dictionaries and equivalents, as well as the gazetteer division, only to adjust and refine the geocoding process.

**Figure 8:**
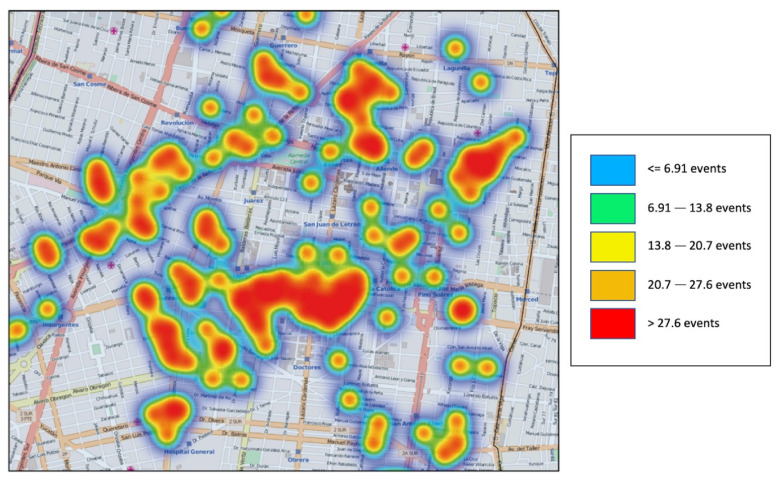
Traffic events represented by a heat map.

In other words, a result is a number of locations where accidents could be placed. Figure 8 shows a representation of correlational traffic events by means of a heat map. This raster representation shows a spatial distribution, which defines the concentration or density of traffic-related events from a specific and sensed area of the Cuauhtémoc District in Mexico City. Additionally, Figure 8 depicts the range of concentration values of the traffic events. These values were obtained by computing all the predictions in rush hours.

### 4.4. Evaluation and Results of the Prediction Model

The regression method works with numeric values that are in the set *R*. Thus, a variation between the predictive locations and the real locations that were used to test the model is proposed. The threshold has been established at 100 m in order to work with coordinates that contain latitude and longitude, which are required to establish how to consider true positives, true negatives, and false negatives values.

In the case of predictions, a *true positive* is considered when an element of the test dataset (gold standard) is inside the threshold of a predicted element by the model. A *true negative* is a predicted element by the model, which is inside the threshold and there is any element of the test dataset. A *false negative* is an element of the test dataset that never was touched by the threshold of any predicted element by the model.

The *precision* and *recall* measures were computed as follows. The test dataset is provided from the training dataset and it is not used to create the model. It is used with the input of the test dataset; the result was compared with the output of the test dataset by using the criterion below in order to consider true positive, true negative, and false negative values.

**Figure 9:**
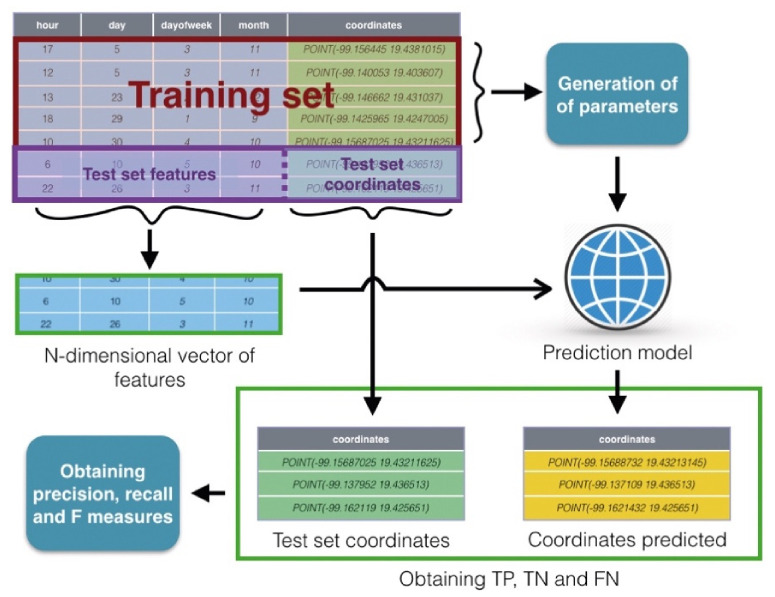
A general schema for the empirical testing of the prediction model.

In Figure 9, a general schema to process the empirical data is presented. Thus, the collection to generate and test the prediction model was divided into two sets called training and test. First, the training set was used to generate the prediction model, considering the empirical data. Moreover, the test set was divided again into inputs (subsets of characteristics vector), and outputs (the obtained coordinates from the geocoding process). The inputs were sent to the SVR model and the obtained output from this model was compared with the output of the test set in order to obtain the true positive (TP), true negative (TN), false positive (FP), and false negative (FN) items.

**Figure 10:**
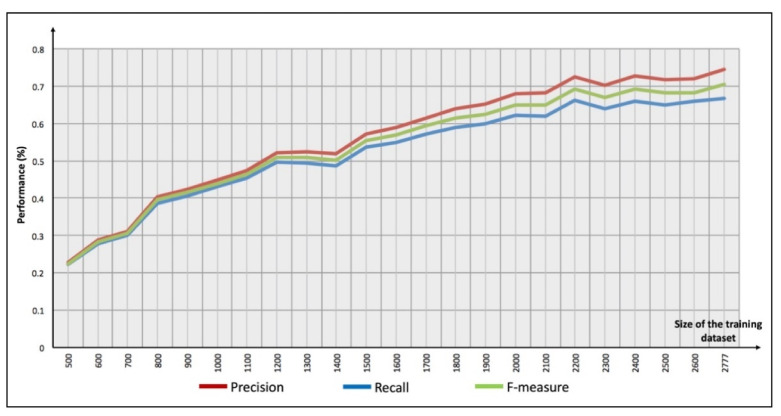
Relationship between the size of the training dataset and the precision, recall, and F-measure.

The first experiment consisted of determining the relationship between the size of the training dataset and the *precision* and *recall* of the created models. By using one district of Mexico City and a threshold of 100 m, several increments were made starting with 500 elements. The *precision* and *recall* increased considerably while adding elements, but the generation of the model increased too; thus, when the training dataset has around 3000 elements, the precision is close to 80% and the time to generate the model is around 1.57 h (see Figure 10).

The second experiment consisted of selecting features, by adding time features such as a minute and second to the initial vector (hour, day, day of the week, month). The results showed that precision and recall fell considerably, from a precision and recall of 70% and 65%, respectively. In addition, they decreased to 47% and 42%, respectively, using a training dataset of 2500 elements. The reason was that by adding these variables, the vector generation was very similar at distant places with others; thus, it produced noise for the model creation.

In fact, the values of the parameters required by the SVR library need to be tested. These values are very relevant to generate the traffic prediction model. Thus, an experiment switching the kernels was carried out. With a linear kernel, there was no solution because it is a non-linear regression problem. Although a polynomial kernel is very useful for Natural Language Processing, it tends to infinity in this kind of problem when the training datasets with more than 20 elements produce a memory overflow. By testing the penalty parameter (*C*), when it has a small value between 0 and 100, the models have low *precision* and *recall* values because the function presents many mistakes in the calibration. Otherwise, with high values of more than 10,000, good results were obtained. However, this fact increased considerably the time to generate the model. Something similar happened with epsilon (ε-tube) because it represents a radius that covers the function, such as a tube; thus, all elements that fell inside this tube were not considered for the learning process. Low values of epsilon ended up overfitting, and high values produced a lack of learning because the radius was very high with respect to the function in which any element did not have the capacity to learn.

## 5. General Discussion

The precision and credibility of the geographic information posted on Twitter can be assessed by three different perspectives. The first one is given by the number of people that can participate in the generation of this information. This effect is also visible in social networks; when an event is exposed by a user account, it is generally accompanied by confirmations made by other followers of the Twitter account without a relationship between them. Thus, if the quantity of users that indicates an event in an isolated way is greater, then this isolated event has a higher weight, and the credibility of that event is real. Regarding the second perspective, it is carried out through the creation of a hierarchy of users and the assignment of roles within the group of users that generate the geographic content. Ref. [48] endorsed that geographic information generation by user accounts follows a distribution with respect to generated information and the number of user accounts; that is, a small group of people generates a large amount of information, and the rest of the user accounts generate moderate information. Thus, to this small group of people a higher role is assigned, which implies additional responsibilities such as information assessment and edition capacity, and among others. The geographic proximity is related to the third approach. Basically, it is the first law of geography *“All things are related to each other, but things that are closer in space have a greater relationship than those that are distant”* [49]. The approach is used to assign a probabilistic value if the exposed event can be true or false. An example is the knowledge of high levels of vehicular traffic on a given avenue; it is highly probable that some record of a vehicular accident is true, and even more so if there have already been records of previous vehicular accidents on this avenue. Thus, these approaches ensure that geographic information generated by users is consistent and with gradual increases in accuracy due to continuous reviews and assessments by the community.

Moreover, a study carried out to assess information on Twitter demonstrated that true information behaves differently from false information. The study identified seven false events, and a tracing of the information behavior on the social network was performed. The results showed that true information is confirmed by 95.5% by the Twitter community and rejected by only 0.3%. On the other hand, false information is rejected by 50% in addition to being questioned by a percentage greater than the confirmation of the event. The study affirms that the Twitter community works as a collaborative filter of information [50]. On the other hand, [27] verified that when Twitter is used during an emergency, other users are not mentioned in the text of the messages. Thus, the percentage varies between 6% and 8% against 22% in a normal situation. Conversely, tweets that include Internet site addresses are higher in an emergency, ranging from 40% to 50% versus 25% in a daily situation. Thus, it is possible to infer that emergencies also follow a pattern that largely validates the veracity of the information.

On the other hand, the generated corpus that represents the new tweet dataset is composed of 65,250 tweets, which were collected over a period of approximately six months, from 7 July 2020 to 22 December 2020. These tweets were cleaning and preprocessing according to the methods described in Section 3.1. Regarding the initial gazetteer, the dataset is composed of 36,236 different streets (more than 150,000 street segments) from Mexico City. It is important to emphasize that the estimation of traffic-related events improves with more collected tweets. Thus, there is no direct relationship among the number of collected data to generate the predictions.

Concerning the case that the prediction model does not receive information from the Twitter stream, the model only generates the computation for the historic and collected data that is stored in the corpus; obviously, the input of the model depends directly on the collected tweet data to increase its precision. However, there is no constraint that the model can continue generating spatiotemporal predictions, but the accuracy of the outcomes could not be appropriate due to the Twitter data stream that is stopped.

## 6. Conclusions and Future Works

Smart cities applications are oriented towards providing better public services and making the use and integration of different computing technology more efficient, such as the Internet of Things, telecommunication networks, wearable sensors, Volunteered Geographic Information, and Machine Learning approaches, among others. Thus, the essential components for urban development in a smart city include smart technology, smart industry, smart services, smart management, and smart life that can be modeled by smart systems or applications.

Many smart city applications have been developed to share road network information. A popular example is Waze, which is a community-based traffic and navigation application. Another example is Traffic Pulse, a participatory mobile sensor web platform that relies on the voluntary use of smartphones to report on road conditions. Thus, the proposed framework could be very useful for mixing and integrating more variables and empirical data from those applications in order to enrich the tweet collection. Thus, the prediction models will improve the precision, analysis, and characterization of the traffic behavior, and other kinds of traffic-related events will be classified efficiently.

The contribution or strengths of the proposed methodology are centered on developing an approach with low cost in which the main collected information is provided by Twitter with an assessment realized by the users of this social network. Thus, the proposed empirical dataset makes a big difference from the conventional traffic data collection methods, which are based on roadside inductive loop detectors that are costly to deploy and maintain. Thus, social media are an alternative that can be leveraged to gather collaboratively Volunteered Geographic Information about the conditions of roads, streets, and avenues. In this direction, this work was intended to design a novel framework to geolocate traffic-related events from the Twitter stream. Thus, the proposed research work is addressed to influence in the crowdsensing field because the benefits not only are focused on developing a methodology to generate prediction models to analyze the traffic congestion, but the geocoding process is also an important key to enrich other contexts and various datasets from different scopes such as air pollution, crime incidence, and human mobility, among others. This takes into consideration human beings as sensors, which represents important opportunities for user-generated content and for facing challenges associated with the dynamic-sensing of cities. 

Moreover, the geocoding method improved considerably by using a gazetteer enriched with information from the Twitter stream. Moreover, an approach to discover how to divide traffic-related events to give more accurate representations is proposed. With this division, it was possible to find the number of geographic elements in a tweet, which could have a relationship with the kind of traffic-related event. It is significantly important to highlight that this research demonstrated that there exists a relationship between Twitter participation and the rush hours in the Mexico City.

According to the predictions, an approach to create training and test datasets from geocoded information is presented. These collections generated a traffic road prediction model as well as an evaluation technique to estimate the model. SVR demonstrated that it is capable to create reliable prediction models regarding traffic-related events. The considerations to keep in mind are the number of elements in the training dataset and the selected features, which are related to the *precision* and *recall* of the model. The SVR parameters are fundamental to create a reliable prediction model in order to avoid overfitting or learning lack.

The geocoding of traffic-related events with training datasets based on an SVR method for making spatiotemporal predictions about the traffic congestions in a specific time is a useful resource to characterize the behavior of the traffic city conditions, such as to how to avoid crowded areas, assign traffic polices, or detect broken traffic lights.

Regarding the weaknesses of the proposed methodology, both the geocoding process and the prediction model present limitations. They are restricted by the conception of their functionality. For example, the weakness of the geocoding process is conceived from the constitution of the gazetteer and the natural conception of the posted tweets. The approach of this methodology does not consider the direction of each event that has occurred due to the gazetteer not containing information related to the direction of the streets. Moreover, many of the tweets do not mention the direction where the traffic event has occurred. For the prediction model, the conceptualization of the Machine Learning method could be imprecise in considering road conditions due to a condition being composed of a coordinate sequence, which would have assigned the same vector of temporal characteristics. This affects considerably the creation of the regression model. Another issue not taken into account is the possible impact of each event on the street, that is, how long an event can affect a road as a baseline; a duration of time t is assigned to each accident when it is predicted or visualized. The amount of information to create the prediction model plays an important role in the implementation because districts that contain few recorded events from the Twitter stream produce poor predictions, but this is not only a limitation of the methodology but also a lack of data that prevents better predictions.

On the other hand, there are applications such as SenseCity that can share open data related to urban participatory and tourist information. It offers an Application Programming Interface (API) for building mobile applications. Thus, smart city systems such as Smart City Vadodara, Enevo, Streetline, Anagog, EverImpact, and TZOA can exchange their sensed information with the proposed framework as an important insight to make predictions that consider traffic-related events, analyzing variables associated with the weather, human mobility, and public transportation networks.

Moreover, many research directions are focused on developing new protocols, architectures, and services that will be devoted to the domain of smart cities. Thus, future works will be oriented towards integrating the information provided from wearable sensors in the context of healthcare applications to measure and analyze people’s stress, glucose level, and assistance for Parkinson’s Disease. Moreover, emergency management, risk mitigation, citizen election engagement, the management of energy consumption, urban public facilities, law enforcement, and air pollution are open issues that big cities are currently facing, and they represent big challenges for the research community.

Regarding improving the geocoding approach, it is important to focus on discovering further relationships among geographic elements and finding other cases that can occur independently of the assumptions established in the present research. At this time, the proposed geocoding method of traffic-related events does not consider the direction of the event. Thus, it is necessary to define an algorithm for inferring the direction where the event is located. The nearest areas outside Mexico City present a lot of urban mobility; thus, expanding this methodology to the nearest areas beyond city boundaries would be a wellness initiative. In fact, a temporal analysis is required to establish a reasonable time duration for an accident or condition, and a baseline could be set up for each traffic-related event.

Moreover, Twitter accounts always post with the same structure; thus, a Machine Learning method could be implemented to learn features, which can be related to the creation of the training dataset, the traffic road congestion model, and its predictions require improvement in different aspects such as the training dataset needing more features to increase the accurate model; a traffic-related event having many relationships, not only temporal but also descriptive attributes such as lane numbers; the number of traffic lights along the way; buildings; and public transportation stations and the neighborhoods near them. By adding these relationships to the training dataset, a better representation of the learning issue could be obtained. The model creation would add a geographic restriction in the SVR method, which handles in a better way the locations that contain the training dataset. Indeed, the precision of results is directly related to the accuracy of the traffic-related events that were previously geocoded; thus, it implies that better geocoded events can produce better predictions.

## Figures and Tables

**Table 1:** Common traffic conditions and events mentioned in the tweet dataset.

Traffic Condition and Event	Number of Occurrences/Count
Emergency service	378
Rollover	612
Accident	1162
Flooding	432
Car crash	1312
Emergency in place	508
Broken car	1002
Vehicular congestion	570
Traffic signals out of service	241
Blocked road	4377
Still close	1053
Heavy traffic	1423
Slow displacement	2779
Road work	1225
Road close	2521
Traffic jam	1423
Bumper to bumper	2246
Gridlock	1101

**Table 2:** The process to find traffic-related events with the relationship (*line*, *line*, *line*).

Pseudocode to Find the Relationship
> While all the possible combinations of elements are not tested: > Is there intersection of *A* element and *B* element: (operation 1) > Save it. > Are there two intersections? > Yes: Is there an element in common from the two intersections? > Yes: Find the bounding box (or convex hull) of the element in common delimited by the two intersections. (operation 3) > No: Check (line, line) relationship. > No: Check (line, line) relationship.

**Table 3:** Selected features and possible values.

Feature	Possible Value	Description
Month	[1,…,12]	Month of the year when a tweet was posted
Day of month	[1,…,31]	Day of the month when a tweet was posted
Day of week	[0,…,6]	Day of the week when the tweet was posted. 0 is for Sunday and 6 for Saturday
Hour of the day	[0,…,23]	Hour of the day when a tweet was posted

**Table 4:** Defined parameters in the SVR library in order to characterize the prediction model.

Parameter	Description	Value
*C*	Penalty parameter *C* of the error term.	1.0
*Epsilon*	It specifies the ε-tube within no penalty and is associated in the training loss function with points predicted within a distance *epsilon* from the actual value.	0.1
*Kernel*	It specifies the kernel type to be used in the algorithm. It must be *linear*, *poly*, *rbf*, *sigmoid*, *precomputed*, or *callable.*	rbf
*Gamma*	Kernel coefficient for *rbf*, *poly*, and *sigmoid*. If *gamma* is automatic, then 1/n features will be used instead.	auto
*Shrinking*	Whether to use the shrinking heuristic.	true
*Tol*	Tolerance for stopping criterion.	1*e*−∍
*Max_iter*	rd limit on iterations within solver, or −1 for no limit.	−1

**Table 5:** Traffic-related Twitter accounts covering Mexico City.

Twitter Account	Geographic Location	Creation Date	Number of Followers	Number of Tweets	Government
Supervia_CDMX	Mexico City	07.14.2010	19,900	29.6 K	Yes
PolloVial	Mexico City	01.31.2013	719	376.6 K	No
Trafico889	Mexico City	05.14.2009	864,500	638.1 K	No
AlerTux	Mexico City	10.16.2012	482,500	623.3 K	No
072AvialCDMX	Mexico City	10.20.2010	221,700	1.5 M	Yes
RedVialRC	Mexico City	03.09.2010	186,500	201.2 K	No

**Table 6:** Results of the evaluation task, applying precision and recall measures.

Description of Elements	Baseline	First Evaluation	Second Evaluation	Third Evaluation	Test Dataset
All elements found	152	152	427	456	652
At least one element found	289	388	599	608	652
Mistakes	363	264	53	44	0.0
Precision	0.39	0.43	0.83	0.85	1.0
Recall	0.31	0.39	0.80	0.83	1.0

## Data Availability

Not applicable.
